# Supplementary material for: HIVprotI: an integrated web based platform for prediction and design of HIV proteins inhibitors
Source: J Cheminform. 2018 Mar 9;10:12. doi: 10.1186/s13321-018-0266-y (PMC5845081; doi:10.1186/s13321-018-0266-y)
Supplement: Supplementary file 2 — Additional file 2. Source code of HIVProtI web server. [file 13321_2018_266_MOESM2_ESM.zip › HIVprotI_Source-code/hivprotI/weka-3-6-6/doc/weka/experiment/Experiment.html]

Experiment


---


|  |  |  |
| --- | --- | --- |
| **Overview** \| **Package** \| **Class** \| **Tree** \| **Deprecated** \| **Index** \| **Help** \| **Weka's home** | |  |
| **PREV CLASS**   **NEXT CLASS** | **FRAMES**    **NO FRAMES**     **All Classes** |  |
| SUMMARY: NESTED \| FIELD \| CONSTR \| METHOD | DETAIL: FIELD \| CONSTR \| METHOD |  |


---


## weka.experiment Class Experiment

```
java.lang.Object
  weka.experiment.Experiment
```

**All Implemented Interfaces:**: java.io.Serializable, OptionHandler, RevisionHandler

**Direct Known Subclasses:**: RemoteExperiment

---

``` public class Experiment extends java.lang.Object implements java.io.Serializable, OptionHandler, RevisionHandler ```

Holds all the necessary configuration information for a standard
type experiment. This object is able to be serialized for storage
on disk.
Valid options are:

```
 -L <num>
  The lower run number to start the experiment from.
  (default 1)
```

```
 -U <num>
  The upper run number to end the experiment at (inclusive).
  (default 10)
```

```
 -T <arff file>
  The dataset to run the experiment on.
  (required, may be specified multiple times)
```

```
 -P <class name>
  The full class name of a ResultProducer (required).
  eg: weka.experiment.RandomSplitResultProducer
```

```
 -D <class name>
  The full class name of a ResultListener (required).
  eg: weka.experiment.CSVResultListener
```

```
 -N <string>
  A string containing any notes about the experiment.
  (default none)
```

```
 Options specific to result producer weka.experiment.RandomSplitResultProducer:
```

```
 -P <percent>
  The percentage of instances to use for training.
  (default 66)
```

```
 -D
 Save raw split evaluator output.
```

```
 -O <file/directory name/path>
  The filename where raw output will be stored.
  If a directory name is specified then then individual
  outputs will be gzipped, otherwise all output will be
  zipped to the named file. Use in conjuction with -D. (default splitEvalutorOut.zip)
```

```
 -W <class name>
  The full class name of a SplitEvaluator.
  eg: weka.experiment.ClassifierSplitEvaluator
```

```
 -R
  Set when data is not to be randomized and the data sets' size.
  Is not to be determined via probabilistic rounding.
```

```
 Options specific to split evaluator weka.experiment.ClassifierSplitEvaluator:
```

```
 -W <class name>
  The full class name of the classifier.
  eg: weka.classifiers.bayes.NaiveBayes
```

```
 -C <index>
  The index of the class for which IR statistics
  are to be output. (default 1)
```

```
 -I <index>
  The index of an attribute to output in the
  results. This attribute should identify an
  instance in order to know which instances are
  in the test set of a cross validation. if 0
  no output (default 0).
```

```
 -P
  Add target and prediction columns to the result
  for each fold.
```

```
 Options specific to classifier weka.classifiers.rules.ZeroR:
```

```
 -D
  If set, classifier is run in debug mode and
  may output additional info to the console
```

All options after -- will be passed to the result producer.

**Version:**
:   $Revision: 5401 $

**Author:**
:   Len Trigg (trigg@cs.waikato.ac.nz)

**See Also:**: Serialized Form

---

| **Field Summary** | |
| --- | --- |
| `static java.lang.String` | `FILE_EXTENSION`             The filename extension that should be used for experiment files |


**Constructor Summary**

`Experiment()`


| **Method Summary** | |
| --- | --- |
| `void` | `advanceCounters()`             Increments iteration counters appropriately. |
| `void` | `classFirst(boolean flag)`             Sets whether the first attribute is treated as the class for all datasets involved in the experiment. |
| `boolean` | `getAdvanceDataSetFirst()`             Get the value of m\_DataSetFirstFirst. |
| `int` | `getCurrentDatasetNumber()`             When an experiment is running, this returns the current dataset number. |
| `int` | `getCurrentPropertyNumber()`             When an experiment is running, this returns the index of the current custom property value. |
| `int` | `getCurrentRunNumber()`             When an experiment is running, this returns the current run number. |
| `javax.swing.DefaultListModel` | `getDatasets()`             Gets the datasets in the experiment. |
| `java.lang.String` | `getNotes()`             Get the user notes. |
| `java.lang.String[]` | `getOptions()`             Gets the current settings of the experiment iterator. |
| `java.lang.Object` | `getPropertyArray()`             Gets the array of values to set the custom property to. |
| `int` | `getPropertyArrayLength()`             Gets the number of custom iterator values that have been defined for the experiment. |
| `java.lang.Object` | `getPropertyArrayValue(int index)`             Gets a specified value from the custom property iterator array. |
| `PropertyNode[]` | `getPropertyPath()`             Gets the path of properties taken to get to the custom property to iterate over. |
| `ResultListener` | `getResultListener()`             Gets the result listener where results will be sent. |
| `ResultProducer` | `getResultProducer()`             Get the result producer used for the current experiment. |
| `java.lang.String` | `getRevision()`             Returns the revision string. |
| `int` | `getRunLower()`             Get the lower run number for the experiment. |
| `int` | `getRunUpper()`             Get the upper run number for the experiment. |
| `boolean` | `getUsePropertyIterator()`             Gets whether the custom property iterator should be used. |
| `boolean` | `hasMoreIterations()`             Returns true if there are more iterations to carry out in the experiment. |
| `void` | `initialize()`             Prepares an experiment for running, initializing current iterator settings. |
| `java.util.Enumeration` | `listOptions()`             Returns an enumeration describing the available options.. |
| `static void` | `main(java.lang.String[] args)`             Configures/Runs the Experiment from the command line. |
| `void` | `nextIteration()`             Carries out the next iteration of the experiment. |
| `void` | `postProcess()`             Signals that the experiment is finished running, so that cleanup can be done. |
| `static Experiment` | `read(java.lang.String filename)`             Loads an experiment from a file. |
| `void` | `runExperiment()`             Runs all iterations of the experiment, continuing past errors. |
| `void` | `setAdvanceDataSetFirst(boolean newAdvanceDataSetFirst)`             Set the value of m\_AdvanceDataSetFirst. |
| `void` | `setDatasets(javax.swing.DefaultListModel ds)`             Set the datasets to use in the experiment |
| `void` | `setNotes(java.lang.String newNotes)`             Set the user notes. |
| `void` | `setOptions(java.lang.String[] options)`             Parses a given list of options. |
| `void` | `setPropertyArray(java.lang.Object newPropArray)`             Sets the array of values to set the custom property to. |
| `void` | `setPropertyPath(PropertyNode[] newPropertyPath)`             Sets the path of properties taken to get to the custom property to iterate over. |
| `void` | `setResultListener(ResultListener newResultListener)`             Sets the result listener where results will be sent. |
| `void` | `setResultProducer(ResultProducer newResultProducer)`             Set the result producer used for the current experiment. |
| `void` | `setRunLower(int newRunLower)`             Set the lower run number for the experiment. |
| `void` | `setRunUpper(int newRunUpper)`             Set the upper run number for the experiment. |
| `void` | `setUsePropertyIterator(boolean newUsePropertyIterator)`             Sets whether the custom property iterator should be used. |
| `java.lang.String` | `toString()`             Gets a string representation of the experiment configuration. |
| `static void` | `write(java.lang.String filename, Experiment exp)`             Writes the experiment to disk. |

**Methods inherited from class java.lang.Object**

`equals, getClass, hashCode, notify, notifyAll, wait, wait, wait`

**Field Detail**

### FILE\_EXTENSION

```
public static java.lang.String FILE_EXTENSION
```

:   The filename extension that should be used for experiment files


**Constructor Detail**

### Experiment

```
public Experiment()
```


**Method Detail**

### classFirst

```
public void classFirst(boolean flag)
```

:   Sets whether the first attribute is treated as the class
    for all datasets involved in the experiment. This information
    is not output with the result of the experiments!

    :   **Parameters:**: `flag` - whether the class attribute is the first and not the last

---


### getAdvanceDataSetFirst

```
public boolean getAdvanceDataSetFirst()
```

:   Get the value of m\_DataSetFirstFirst.

    :   **Returns:**: Value of m\_DataSetFirstFirst.

---


### setAdvanceDataSetFirst

```
public void setAdvanceDataSetFirst(boolean newAdvanceDataSetFirst)
```

:   Set the value of m\_AdvanceDataSetFirst.

    :   **Parameters:**: `newAdvanceDataSetFirst` - Value to assign to m\_AdvanceRunFirst.

---


### getUsePropertyIterator

```
public boolean getUsePropertyIterator()
```

:   Gets whether the custom property iterator should be used.

    :   **Returns:**: true if so

---


### setUsePropertyIterator

```
public void setUsePropertyIterator(boolean newUsePropertyIterator)
```

:   Sets whether the custom property iterator should be used.

    :   **Parameters:**: `newUsePropertyIterator` - true if so

---


### getPropertyPath

```
public PropertyNode[] getPropertyPath()
```

:   Gets the path of properties taken to get to the custom property
    to iterate over.

    :   **Returns:**: an array of PropertyNodes

---


### setPropertyPath

```
public void setPropertyPath(PropertyNode[] newPropertyPath)
```

:   Sets the path of properties taken to get to the custom property
    to iterate over.

    :   **Parameters:**: `newPropertyPath` - an array of PropertyNodes

---


### setPropertyArray

```
public void setPropertyArray(java.lang.Object newPropArray)
```

:   Sets the array of values to set the custom property to.

    :   **Parameters:**: `newPropArray` - a value of type Object which should be an array of the appropriate values.

---


### getPropertyArray

```
public java.lang.Object getPropertyArray()
```

:   Gets the array of values to set the custom property to.

    :   **Returns:**: a value of type Object which should be an array of the appropriate values.

---


### getPropertyArrayLength

```
public int getPropertyArrayLength()
```

:   Gets the number of custom iterator values that have been defined
    for the experiment.

    :   **Returns:**: the number of custom property iterator values.

---


### getPropertyArrayValue

```
public java.lang.Object getPropertyArrayValue(int index)
```

:   Gets a specified value from the custom property iterator array.

    :   **Parameters:**: `index` - the index of the value wanted **Returns:**: the property array value

---


### getCurrentRunNumber

```
public int getCurrentRunNumber()
```

:   When an experiment is running, this returns the current run number.

    :   **Returns:**: the current run number.

---


### getCurrentDatasetNumber

```
public int getCurrentDatasetNumber()
```

:   When an experiment is running, this returns the current dataset number.

    :   **Returns:**: the current dataset number.

---


### getCurrentPropertyNumber

```
public int getCurrentPropertyNumber()
```

:   When an experiment is running, this returns the index of the
    current custom property value.

    :   **Returns:**: the index of the current custom property value.

---


### initialize

```
public void initialize()
                throws java.lang.Exception
```

:   Prepares an experiment for running, initializing current iterator
    settings.

    :   **Throws:**: `java.lang.Exception` - if an error occurs

---


### hasMoreIterations

```
public boolean hasMoreIterations()
```

:   Returns true if there are more iterations to carry out in the experiment.

    :   **Returns:**: true if so

---


### nextIteration

```
public void nextIteration()
                   throws java.lang.Exception
```

:   Carries out the next iteration of the experiment.

    :   **Throws:**: `java.lang.Exception` - if an error occurs

---


### advanceCounters

```
public void advanceCounters()
```

:   Increments iteration counters appropriately.

---


### runExperiment

```
public void runExperiment()
```

:   Runs all iterations of the experiment, continuing past errors.

---


### postProcess

```
public void postProcess()
                 throws java.lang.Exception
```

:   Signals that the experiment is finished running, so that cleanup
    can be done.

    :   **Throws:**: `java.lang.Exception` - if an error occurs

---


### getDatasets

```
public javax.swing.DefaultListModel getDatasets()
```

:   Gets the datasets in the experiment.

    :   **Returns:**: the datasets in the experiment.

---


### setDatasets

```
public void setDatasets(javax.swing.DefaultListModel ds)
```

:   Set the datasets to use in the experiment

    :   **Parameters:**: `ds` - the list of datasets to use

---


### getResultListener

```
public ResultListener getResultListener()
```

:   Gets the result listener where results will be sent.

    :   **Returns:**: the result listener where results will be sent.

---


### setResultListener

```
public void setResultListener(ResultListener newResultListener)
```

:   Sets the result listener where results will be sent.

    :   **Parameters:**: `newResultListener` - the result listener where results will be sent.

---


### getResultProducer

```
public ResultProducer getResultProducer()
```

:   Get the result producer used for the current experiment.

    :   **Returns:**: the result producer used for the current experiment.

---


### setResultProducer

```
public void setResultProducer(ResultProducer newResultProducer)
```

:   Set the result producer used for the current experiment.

    :   **Parameters:**: `newResultProducer` - result producer to use for the current experiment.

---


### getRunUpper

```
public int getRunUpper()
```

:   Get the upper run number for the experiment.

    :   **Returns:**: the upper run number for the experiment.

---


### setRunUpper

```
public void setRunUpper(int newRunUpper)
```

:   Set the upper run number for the experiment.

    :   **Parameters:**: `newRunUpper` - the upper run number for the experiment.

---


### getRunLower

```
public int getRunLower()
```

:   Get the lower run number for the experiment.

    :   **Returns:**: the lower run number for the experiment.

---


### setRunLower

```
public void setRunLower(int newRunLower)
```

:   Set the lower run number for the experiment.

    :   **Parameters:**: `newRunLower` - the lower run number for the experiment.

---


### getNotes

```
public java.lang.String getNotes()
```

:   Get the user notes.

    :   **Returns:**: User notes associated with the experiment.

---


### setNotes

```
public void setNotes(java.lang.String newNotes)
```

:   Set the user notes.

    :   **Parameters:**: `newNotes` - New user notes.

---


### listOptions

```
public java.util.Enumeration listOptions()
```

:   Returns an enumeration describing the available options..

    :   **Specified by:**: `listOptions` in interface `OptionHandler`
    :   **Returns:**: an enumeration of all the available options.

---


### setOptions

```
public void setOptions(java.lang.String[] options)
                throws java.lang.Exception
```

:   Parses a given list of options.


    Valid options are:

    ```
     -L <num>
      The lower run number to start the experiment from.
      (default 1)
    ```

    ```
     -U <num>
      The upper run number to end the experiment at (inclusive).
      (default 10)
    ```

    ```
     -T <arff file>
      The dataset to run the experiment on.
      (required, may be specified multiple times)
    ```

    ```
     -P <class name>
      The full class name of a ResultProducer (required).
      eg: weka.experiment.RandomSplitResultProducer
    ```

    ```
     -D <class name>
      The full class name of a ResultListener (required).
      eg: weka.experiment.CSVResultListener
    ```

    ```
     -N <string>
      A string containing any notes about the experiment.
      (default none)
    ```

    ```
     Options specific to result producer weka.experiment.RandomSplitResultProducer:
    ```

    ```
     -P <percent>
      The percentage of instances to use for training.
      (default 66)
    ```

    ```
     -D
     Save raw split evaluator output.
    ```

    ```
     -O <file/directory name/path>
      The filename where raw output will be stored.
      If a directory name is specified then then individual
      outputs will be gzipped, otherwise all output will be
      zipped to the named file. Use in conjuction with -D. (default splitEvalutorOut.zip)
    ```

    ```
     -W <class name>
      The full class name of a SplitEvaluator.
      eg: weka.experiment.ClassifierSplitEvaluator
    ```

    ```
     -R
      Set when data is not to be randomized and the data sets' size.
      Is not to be determined via probabilistic rounding.
    ```

    ```
     Options specific to split evaluator weka.experiment.ClassifierSplitEvaluator:
    ```

    ```
     -W <class name>
      The full class name of the classifier.
      eg: weka.classifiers.bayes.NaiveBayes
    ```

    ```
     -C <index>
      The index of the class for which IR statistics
      are to be output. (default 1)
    ```

    ```
     -I <index>
      The index of an attribute to output in the
      results. This attribute should identify an
      instance in order to know which instances are
      in the test set of a cross validation. if 0
      no output (default 0).
    ```

    ```
     -P
      Add target and prediction columns to the result
      for each fold.
    ```

    ```
     Options specific to classifier weka.classifiers.rules.ZeroR:
    ```

    ```
     -D
      If set, classifier is run in debug mode and
      may output additional info to the console
    ```

    All options after -- will be passed to the result producer.

    :   **Specified by:**: `setOptions` in interface `OptionHandler`
    :   **Parameters:**: `options` - the list of options as an array of strings **Throws:**: `java.lang.Exception` - if an option is not supported

---


### getOptions

```
public java.lang.String[] getOptions()
```

:   Gets the current settings of the experiment iterator.

    :   **Specified by:**: `getOptions` in interface `OptionHandler`
    :   **Returns:**: an array of strings suitable for passing to setOptions

---


### toString

```
public java.lang.String toString()
```

:   Gets a string representation of the experiment configuration.

    :   **Overrides:**: `toString` in class `java.lang.Object`
    :   **Returns:**: a value of type 'String'

---


### read

```
public static Experiment read(java.lang.String filename)
                       throws java.lang.Exception
```

:   Loads an experiment from a file.

    :   **Parameters:**: `filename` - the file to load the experiment from **Returns:**: the experiment **Throws:**: `java.lang.Exception` - if loading fails

---


### write

```
public static void write(java.lang.String filename,
                         Experiment exp)
                  throws java.lang.Exception
```

:   Writes the experiment to disk.

    :   **Parameters:**: `filename` - the file to write to: `exp` - the experiment to save **Throws:**: `java.lang.Exception` - if writing fails

---


### main

```
public static void main(java.lang.String[] args)
```

:   Configures/Runs the Experiment from the command line.

    :   **Parameters:**: `args` - command line arguments to the Experiment.

---


### getRevision

```
public java.lang.String getRevision()
```

:   Returns the revision string.

    :   **Specified by:**: `getRevision` in interface `RevisionHandler`
    :   **Returns:**: the revision


---


|  |  |  |
| --- | --- | --- |
| **Overview** \| **Package** \| **Class** \| **Tree** \| **Deprecated** \| **Index** \| **Help** \| **Weka's home** | |  |
| **PREV CLASS**   **NEXT CLASS** | **FRAMES**    **NO FRAMES**     **All Classes** |  |
| SUMMARY: NESTED \| FIELD \| CONSTR \| METHOD | DETAIL: FIELD \| CONSTR \| METHOD |  |


---
